# Editorial: Women in antimicrobials, resistance and chemotherapy: 2023

**DOI:** 10.3389/fmicb.2024.1463728

**Published:** 2024-08-06

**Authors:** Cindy Shuan Ju Teh, Krassimira R. Hristova, Ana R. Freitas

**Affiliations:** ^1^Department of Medical Microbiology, Faculty of Medicine, Universiti Malaya, Kuala Lumpur, Malaysia; ^2^Department of Biological Sciences, Marquette University, Milwaukee, WI, United States; ^3^UCIBIO, Applied Molecular Biosciences Unit, Laboratory of Microbiology, Department of Biological Sciences, Faculty of Pharmacy, University of Porto, Porto, Portugal; ^4^Associate Laboratory i4HB, Institute for Health and Bioeconomy, Faculty of Pharmacy, University of Porto, Porto, Portugal; ^5^1H-TOXRUN – One Health Toxicology Research Unit, University Institute of Health Sciences, CESPU, CRL, Gandra, Portugal

**Keywords:** women's empowerment, antimicrobial resistance, antimicrobial activity, virulence, microbiota, alternative drug therapies

In honor of International Women's Day 2023, Frontiers in Microbiology launched the second edition of the “*Women in antimicrobials, resistance and chemotherapy*” Research Topic. This dedicated Frontiers Research Topic was entirely curated by women editors, celebrating their remarkable contributions in this field. It's heartening to see such recognition, especially given the historical undervaluation of women's achievements in STEM (science, technology, engineering, and mathematics). Notable women like Ada Lovelace, Rosalind Franklin, Barbara McClintock, and Marie Curie have left indelible marks on scientific progress, yet gender disparities persist (2021).

Gender and antimicrobial resistance (AMR) also intersect. A recent global review by the World Health Organization (WHO) revealed that women might be more susceptible to developing drug-resistant infections compared to men (Wong, 2024). This gender-specific aspect of AMR remains under-recognized, with over 70% of countries failing to acknowledge gender inequalities in their national plans to combat drug-resistant infections. Approximately 20% of these studies focused on Africa, and nearly 15% focused on southeast Asia. In regions with limited access to clean water, women and girls are at greater risk of drug-resistant urinary tract infections due to menstrual-hygiene needs. Additionally, women, who constitute 70% of global health-care workers, are more likely to encounter drug-resistant infections in hospitals and clinics. Higher rates of sexual violence against women also increase their vulnerability to drug-resistant sexually transmitted infections. And if progress on empowering women and girls was already too slow, COVID-19 and the war in Ukraine and Gaza have exacerbated entrenched gender inequalities. In this sense, UN Women's Strategic Plan 2022–2025 centers the activity of women and girls, guiding work across normative spheres, UN coordination, and program implementation, tailored to national contexts and executed with various partners to support women and girls in creating a better future (United Nations, 2021).

The papers published on this Research Topic (8 original Research Articles, one Review, one errata) shed light on the vital work of female researchers addressing antimicrobial resistance—one of the top global health threats of our time.

Thurner and Alatraktchi critically reviewed the role of sub-lethal antibiotics in the evolution of bacterial virulence. Besides the mutations and natural selection, alteration in virulence could also be achieved through enrichment in sub-Minimum Inhibitory Concentration (MIC). To further study how virulence manifests itself under sub-MIC, methodologies for sub-MIC determination and measurement of virulence should be standardized. This will provide useful information for patient management.

Barone et al. investigated the effects of HDAC6 inhibitors, which act on histone deacetylation and bacterial clearance, against *Pseudomonas aeruginosa*. The results showed changes in the production of virulence factors like pyocyanin and rhamnolipids. Selective HDAC6 inhibitors demonstrated potential in reducing inflammation and bacterial load in chronic infection models mimicking the cystic fibrosis (CF) phenotype. However, further research is needed to understand the molecular mechanisms of HDAC6's role in bacterial infections.

Kanafani et al. aimed at exploring the differential activity of ciprofloxacin and levofloxacin on the selection of resistance among 233 clinical isolates of *Pseudomonas aeruginosa*. Levofloxacin required fewer passages than ciprofloxacin to reach breakpoints in some isolates, and induced mutants against both drugs had higher fitness costs compared to parental isolates. Whole genome sequencing revealed alterations in *gyrA, gyrB*, and *parC* genes associated with resistance.

Li et al. described the *in vitro* antibacterial activity of novel β-lactam-β-lactamase inhibitor combinations (BLBLIs) against carbapenem-resistant *Klebsiella pneumoniae* (CRKP) producing metallo- and serine-β-lactamases. Using broth microdilution and time-kill assays, they demonstrated significant bactericidal activity for aztreonam combined with new BLBLIs. However, further studies including more isolates with different carbapenemases are needed for comprehensive susceptibility data.

Xu et al. reported the changes of the gut microbiota in children who received broad-spectrum antibiotics in pediatric intensive care unit (PICU). After the treatment, gene functions of the gut microbiota were altered, mainly in those responsible for metabolism, DNA catabolism, and transmembrane transport. Ninety resistance genes were significantly upregulated in Day 7 compared to Day 1. This study has provided a substantial basis for a better understanding of the structure and function of gut microbiota after the short-term admission to PICU.

Jordão et al. investigated the combination of DNase I enzyme with antimicrobial photodynamic therapy (aPDT) in mice infected with fluconazole-susceptible (CaS) and -resistant (CaR) *Candida albicans* strains. After inoculation, the mice received DNase treatment followed by photosensitizer (PDZ) and light exposure for 5 consecutive days. The combination of DNase with PDZ-aPDT significantly reduced fungal viability in mice tongues, with remission of oral lesions observed after 7 days. This approach holds promise for improving the efficacy of photodynamic treatment.

Ye et al. described a green synthesis method for silver nanoparticles using *Cnidium monnieri* fruit extract, highlighting its simplicity, safety, and environmental friendliness compared to physical and chemical methods. These CM-AgNPs demonstrated antifungal activity against Trichophyton rubrum, *T. mentagrophytes*, and Candida albicans, showing potential for treating fungal infections in humans and animals. Developing new topical antifungal drugs with these green CM-AgNPs could be an effective alternative for managing multidrug-resistant candida and dermatophyte infections.

Kasozi et al. surveyed rural community of livestock farmers to determine whether appropriate practices were used in administration of drugs against animal African trypanosomiasis (AAT) in southwestern Uganda. The survey involved farmers and “professionals” i.e., livestock extension officers and drug shop attendants. On around two-thirds of farms, trypanocidal drugs were being administered by farmers and almost all drugs were obtained from privately-owned drug shops. Farmers were more likely than professionals to use antibiotics as well as trypanocidal drugs to treat AAT, raising concerns as to overuse and misuse of antibiotics. The study also highlights the complexity of issues involved in the fight against AAT using drug treatment, and the importance of best farming practices to reduce the emergence of resistant strains of trypanosomes.

Wiesner-Friedman et al., used the microbial Find, Inform, and Test framework to identify strong associations between sources of antimicrobial resistance (AMR), represented by a panel of 5 ARGs/MGEs. Land application of waste (e.g., dairy manure, biosolids) and dairy concentrated animal feeding operations were indicated as important sources of elevated AMR levels in riverbed sediment and surface water. While the consistency in the types of sources selected is strong, differences in estimated overland influence ranges and database types were noted. Overall, this work indicates offsite migration of wastes and calls for more fine-spatial and temporal studies of AMR in watersheds.

Collectively, these investigations showcase the exceptional quality of research led by diverse women scientists, addressing one of the top ten global public health threats. We hope these articles will inspire readers working in the AMR field to explore and tackle AMR from multifaceted perspectives.

## Author contributions

CT: Methodology, Writing – original draft, Writing – review & editing. KH: Methodology, Writing – original draft, Writing – review & editing. AF: Methodology, Writing – original draft, Writing – review & editing, Conceptualization.
